# *iMAP*ping the Perturb-Atlas

**DOI:** 10.1093/lifemedi/lnac057

**Published:** 2022-11-30

**Authors:** Yiwen Sun, Wenyang Lin, Ravinder Kaundal, Tian Chi

**Affiliations:** Gene Editing Center, School of Life Sciences and Technology, ShanghaiTech University, Shanghai 201210, China; Gene Editing Center, School of Life Sciences and Technology, ShanghaiTech University, Shanghai 201210, China; Department of Immunobiology, Yale University School of Medicine, New Haven, CT 06520, USA; Department of Pharmacology and Toxicology, National Institute of Pharmaceutical Education and Research Raebareli (NIPER-R), Lucknow, UP 226002, India; Gene Editing Center, School of Life Sciences and Technology, ShanghaiTech University, Shanghai 201210, China; Department of Immunobiology, Yale University School of Medicine, New Haven, CT 06520, USA

## Abstract

A key objective of the research in the postgenomic era is to decipher the functions of the mammalian genome, which has remained largely enigmatic despite intensive efforts in the functional genomics field over the past two decades. To attack this problem, we have combined the CRISPR-Cas and Cre-Lox technologies to develop iMAP (inducible Mosaic Animal for Perturbation), a transformative tool for rapidly unraveling mammalian genome function *in situ*. Furthermore, we have used iMAP to rapidly construct a “Perturb-Atlas” profiling the functions of 90 protein-coding genes across 39 tissues in mice, which has offered rich insights into gene functions difficult to readily obtain using conventional mouse gene-targeting models. In this research highlight, we offer a brief primer on the iMAP technology, outlining its mechanism, strengths and limitations, and pointing out future directions of research in the area.

Humans have about 20,000 protein-coding genes and over 500 types of cells. Although the sequences of these genes have been known for over 20 years, their functions in various cell types remain largely enigmatic, which has become a key obstacle to disease diagnosis and treatment. Thus, a major objective of the post-genomic biomedical research is to decipher the functions of the protein-coding genes [1]. An effective strategy to achieve this goal would be to perturb the expression of each of these genes in each of the cell types and characterize the direct, cell-intrinsic effects of the perturbations on the cells (in terms of cell survival, proliferation, transcriptome, and epigenome, etc), namely to map the “Perturb-Atlas” (PA). Unfortunately, even in the mouse, the foremost model mammal where the genetic tools are more sophisticated than in other mammalian models, the existing gene-targeting methods are hardly applicable to PA, including single-gene knockout (KO), *in vivo* CRISPR screening based on viral sgRNA libraries, and chemical- or transposon-based random mutagenesis. Thus, gene perturbation methods suitable for PA mapping remain elusive.

The above-mentioned predicament in functional genomics has now been much alleviated with the advent of inducible Mosaic Animal for Perturbation (iMAP), a large-scale multiplexed mosaic CRISPR perturbation method we recently reported, which enables one to disrupt and study ~100 genes in parallel in mice [2]. iMAP is built on the CRISPR-Cas9 and the Cre-lox technologies. At its heart is a transgene carrying up to 100 individually floxed, tandemly linked sgRNA expression units, placed under the control of the U6 promoter (Fig. 1A). Normally, only the first sgRNA (located immediately adjacent the U6 promoter) is expressed. However, following CreER-mediated stochastic recombination of the transgene, triggered by the administration of the small-molecule compound Tamoxifen (TAM), all the downstream sgRNAs on the transgene can be induced in the mice, but only one of these per cell (Fig. 1B), which would subsequently disrupt the corresponding target genes in the presence of Cas9 (Fig. 1C). When both CreER and Cas9 are ubiquitously expressed, TAM converts the mice into mosaic organisms suitable for mapping the PA across diverse mouse tissues. Furthermore, since the sperm also carries the perturbations, the mosaic males can readily sire many conventional single-perturbation lines (Fig. 1D), which greatly reduces the cost of their production; such lines have complementary utilities to the mosaic mice, such as the validation and in-depth study of the candidate genes emerging in the mosaic mice and the genetic screens for organism-level phenotypes.

**Figure 1.**
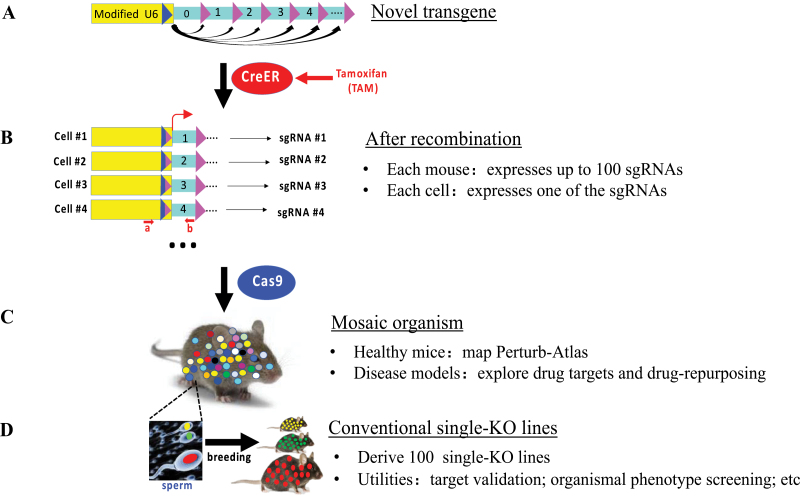
Principle of iMAP. (A) Transgene before recombination. The blue and pink triangles represent a pair of LoxP variants that can undergo only one round of productive recombination (black arrows), which is essential for preventing sequential sgRNA expression within the same cells. The transgene is flanked by PiggyBac ITR (not shown) to enable single-copy random insertion into the mouse genome. (B) The transgene after recombination. a/b is a PCR primer pair for amplifying all expressed sgRNAs before NGS. (C) Mice after Cas9-mediated perturbation. The colored dots represent individual cells with various genes targeted. (D) Derivation of single-gene KO lines from the mosaic fathers.

iMAP has multiple appealing features. First, iMAP is technically straightforward, with perturbation achieved simply by TAM administration. Second, since the targeting is mosaic, with a particular gene disrupted only in a tiny fraction of the target cell population, cell-extrinsic effects that would confound the analysis of cell-intrinsic effects are rare. Third, the results from iMAP analysis are physiologically relevant, because the perturbation is performed *in situ*, with the cells living in their native environment. Fourth, iMAP is versatile in that diverse tissues are amenable to perturbation, assuming Cas9 is effective in these tissues. Fifth, iMAP is scalable (through the creation of more transgenic lines). Finally, like conventional transgenic mice, the iMAP lines can be maintained, expanded, and distributed as permanent community resources, an important advantage if the iMAP mice are needed in large quantities or over time.

In the recent paper [2], we first sought to prove the principle of iMAP by generating an iMAP line targeting six genes with well-defined, readily detectable functions. The six genes fall into three categories (two genes per category): essential genes, tumor suppressor genes, and cell surface receptor genes, whose KO would lead to cell death, cell proliferation, and loss of cell surface markers, respectively. 10 sgRNAs were designed per gene, the abundance of each quantified across 10 major organs/tissues/cell types (termed tissues hereafter) in the induced mosaic adults. Our results revealed the robustness of iMAP, and also indicated that the sgRNAs computationally designed were mostly active and specific, suggesting that the one (instead of 10) sgRNA per gene would be largely sufficient. We have thus adopted this configuration in subsequent experiments to maximize the iMAP throughput.

We next sought to use iMAP to construct a miniature version of the PA. To this end, we created an iMAP line targeting 90 genes whose functions are mostly unclear, and measured the sgRNA abundance across 39 tissues in the mosaic adults. The results offer rich information about gene functions that are difficult to readily obtain using conventional gene-targeting models. In particular, we found 12 sgRNAs depleted across all the 39 tissues, indicating that their target genes are the “core essential genes” (CEGs) generally required for cell survival/proliferation [3]. CEGs, of interest to synthetic biology and cancer biology, have been discovered mainly by CRISPR screening in tumor cell lines, but iMAP enables their systematic discoveries under the physiological condition, namely, in the normal (primary) cells in healthy mice. We also found many genes with tissue-specific functions. For example, *Hdac7* sgRNA was severely depleted in naive CD8 T cells but significantly recovered in effector/memory CD8 T cells, suggesting that *Hdac7* KO promotes CD8 T cell activation, and that knocking out the human ortholog might potentiate human anti-tumor CAR-T cells, the latter we indeed observed. These data demonstrate the power of iMAP in mapping the PA.

The future directions are multifarious. First, like all novel technologies, iMAP needs optimization, particularly in terms of throughput (currently 100 target genes per line) and perturbation efficiency (currently low in postmitotic cells, which seems an intrinsic limitation of Cas9). Second, we have used the bulk sgRNA abundance as the readout for iMAP perturbation. However, iMAP is theoretically amenable to single-cell transcriptomic and epigenomic measurements, which would be far more informative than the bulk sgRNA abundance [4]. A comprehensive pan-tissue whole-genome single-cell PA would be a watershed in the history of biomedical research, because the PA would enable gene function discoveries with the search engine, much like AlphaFold does to protein structures. Third, iMAP can also be coupled to other formats of CRISPR perturbation beside CRISPR editing, including CRISPR interference, CRISPR activation, and Cas13-mediated RNA degradation, the latter ideal for interrogating the function of the non-coding RNAs [5, 6]. Fourth, in addition to systematic mapping of the PA in healthy mice, iMAP performed in disease models should be useful for discovering drug targets and for repurposing old drugs. Finally, iMAP may be applicable to other multicellular organisms beside mice, including plants.
